# Impact of the Covid pandemic on the mental health of children and young people with pre-existing mental health and neurodevelopmental conditions: a systematic review and meta-analysis

**DOI:** 10.1192/j.eurpsy.2024.315

**Published:** 2024-08-27

**Authors:** B. C. F. Ching, V. Parlatini, S. Zhang, H. Abdul Cader, J. Penhallow, E. Voraite, T. Popnikolova, A. Wickersham, J. Downs, E. Simonoff

**Affiliations:** King’s College London, London, United Kingdom

## Abstract

**Introduction:**

Existing systematic reviews have suggested mixed effects of the Covid pandemic on the mental health of children and young people. Those with pre-existing mental health and neurodevelopmental conditions have been suggested to be disproportionately affected, but this has not been meta-analysed. Most reviews of studies in clinical populations to date only include cross-sectional studies during the first lockdown or longitudinal cohorts up to early 2021, which limits our understanding of causality and long-term effects. To our knowledge, this is the first systematic review and meta-analysis to examine the longitudinal impact of the pandemic on the mental health of children and young people with pre-existing mental health and neurodevelopmental conditions.

**Objectives:**

To compare 1) mental health pre versus during Covid, and 2) mental health during Covid.

**Methods:**

Medline, Embase, APA PsycInfo, and Global Health databases were searched up to August 2023. Longitudinal studies reporting mental health outcomes in children and young people (≤18 years old) with pre-Covid mental health and/or neurodevelopmental conditions were included. Cohorts were deemed eligible if children and young people were diagnosed using a diagnostic assessment, scored above clinical threshold on validated measures, or attended mental health services pre-Covid. Outcomes included internalising, externalising, and other symptoms. Studies were narratively synthesised by symptom category and meta-analyses performed where number of studies reporting the same outcomes were sufficient (≥5).

**Results:**

6,083 records were identified and 21 studies (N=2,617) were included. These widely differed in country, setting, diagnosis, outcome, and timepoints under study. The narrative synthesis highlighted mixed findings in mental health changes during the pandemic for all three symptom categories showing increases, reductions, and no changes. Only studies reporting changes in internalising symptoms pre- versus during the pandemic were in sufficient number to be amenable to meta-analysis.

**Conclusions:**

Our findings suggest the pandemic’s impact on the mental health of children and young people with pre-existing mental health and neurodevelopmental conditions were complex and varied. We highlight an urgent need for longitudinal Covid research on long-term mental health outcomes in this vulnerable group. Understanding risk factors and longitudinal trajectories is warranted to guide clinical practice and policy.

**Disclosure of Interest:**

None Declared

